# H-type congenital tracheo-oesophageal fistula associated with oesophageal stenosis: anatomical variant

**DOI:** 10.1093/icvts/ivab343

**Published:** 2021-11-29

**Authors:** Bing Li, Shi-Ting Li, Wei-Bing Chen, Shun-Lin Xia

**Affiliations:** 1 Department of Pediatric Surgery, Huai’an Women and Children’s Hospital, Jiangsu, China; 2 Department of Thoracic surgery, The Affiliated Huai’an No 1 People’s Hospital of Nanjing Medical University, Jiangsu, China

**Keywords:** H-type tracheo-oesophageal fistula, Newborn infant, Stenosis, Thoracoscopy

## Abstract

The goal of this paper was to report a new variant of oesophageal atresia: an H-type congenital tracheo-oesophageal fistula associated with oesophageal segmental stenosis distal to the fistula. Although symptoms were present from birth, we did not differentiate the new anatomical variant preoperatively. The patient was treated by fistula ligation, segmental resection of the distal oesophagus and end-to-end anastomosis of the oesophagus by thoracoscopic surgery. Here we describe the clinical history and management of the newborn infant, together with diagnostic recommendations to prevent misdiagnosis in the management of this condition.

## CASE REPORT

The female infant was born at 39 weeks by emergency caesarean delivery at her local hospital with a birth weight of 3.5 kg. Shortly thereafter, the infant developed tachypnoea, cyanosis and excessive salivation; oesophageal atresia (EA) was suspected. The orogastric intubation was unsuccessful. Chest and abdominal radiographs were performed confirming that the nasogastric tube was lodged at the level of T3 with air identified in the stomach and intestine. A thoracic roentgenogram visualized the proximal blind end of the oesophagus: The diagnosis of type IIIb EA was made. A plain X-ray film of the chest showed contrast aspiration in bilateral bronchi and lungs after the oesophagram. Because we thought the patient had a type IIIb EA, we did not perform bronchoscopy.

### Ethics statement

This study protocol was approved by the ethics committee of the Huai’an Children’s Hospital (Jiangsu, China). Informed consent was obtained from the parents before the operation.

All relevant data are within the manuscript and its supporting information files.

**Figure 1:**
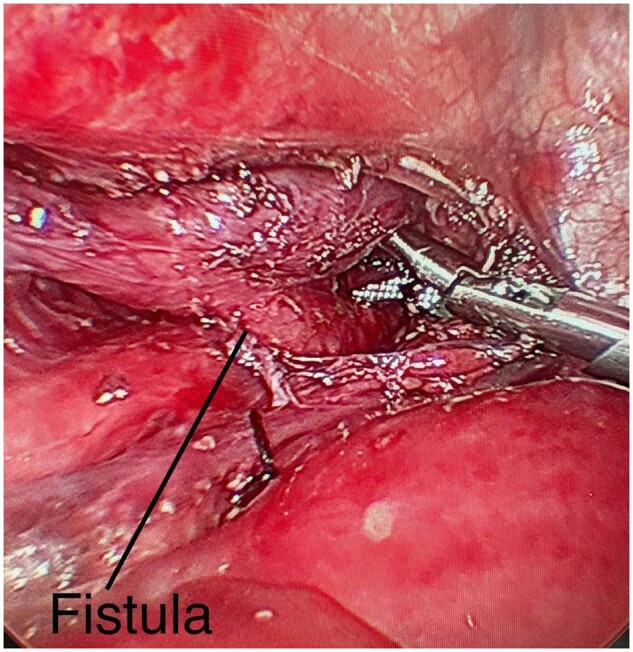
A fistula in an oblique course located between the posterior wall of the trachea and the anterior wall of the oesophagus.

The operation was performed by thoracoscopic surgery on day 1 of life. The infant was placed in a prone position on the table, with the right chest elevated 45°. The azygos vein was mobilized and ligated. Careful separation of the oesophagus was performed, but neither a distal fistula nor a proximal blind end was detected. The oesophageal wall was intact, which meant no atresia in the oesophagus. As we continued the upward mobilization, we detected an abnormal communication in an oblique course located between the posterior wall of the trachea and the anterior wall of the oesophagus in the thoracic inlet (Fig. 1). Then we made the diagnosis of an H-type congenital tracheo-oesophageal fistula. The fistula was divided, then ligated with 4–0 silk suture near the trachea and cut off at the side of the oesophagus. The fistula communicated to the oesophagus, and the oesophagus was obstructed distal to the fistula. After cutting off the distal part of the oesophagus longitudinally, we identified an 8-mm long strictured segment of the oesophagus. The strictured segment was removed. Then an oesophageal end-to-end anastomosis was completed with 5–0 polydioxanone sutures with a size 6-Fr transanastomotic tube left in situ. Finally, the H-type congenital tracheo-oesophageal fistula associated with distal oesophageal stenosis was diagnosed.

A subsequent oesophagram on postoperative 7 revealed normal motility of the oesophagus without leakage. Oral feeding was commenced on postoperative day 8, and the baby was discharged on postoperative day 14. The postoperative period was uneventful.

## DISCUSSION

Five anatomical types of EA had been reported; congenital tracheo-oesophageal fistula without EA (H-type) accounts for about 4% of these cases [1, 2]. Some variations of EA have been described in clinics [3]. Congenital oesophageal stenosis is a type of oesophageal stenosis that could be isolated or associated with EA. Three histological subtypes (tracheobronchial remnants, fibromuscular thickening and membranous webbing) have been described in the literature [4].

Our case is anatomically similar to the common form of EA with a distal tracheo-oesophageal fistula. In cases with type C, the fistula entered the distal oesophagus, and the proximal and distal segments of the oesophagus separated completely [5]. Even though in some cases the 2 blind ends were extremely close, their walls were anatomically separated. Furthermore, the fistula never entered the proximal oesophagus. In our case, neither a distal fistula nor a proximal blind end was detected. The oesophageal wall was anatomically intact, with an abnormal communication located between the posterior wall of the trachea and the anterior wall of the oesophagus that led to a diagnosis of an H-type congenital tracheo-oesophageal fistula. The proximal pouch actually did exist in the preoperative oesophagram, making discrimination of the tracheo-oesophageal fistula and the distal oesophageal stricture more difficult. The fistula entered the proximal oesophagus; the proximal and distal oesophagus had a small hole to communicate, allowing air to enter the proximal and distal oesophagus, and air could still enter the belly.

For the cases of a Y-type fistula [3], both the distal and proximal pouches had a fistula, and the 2 fistulas connected to a Y-shaped configuration and then entered the trachea. The distal and proximal pouches separated anatomically. But our patient had an intact oesophageal wall with 1 fistula connected with the trachea. This configuration could not be discriminated clearly by the thoracoscopic approach.

Most of the type III EAs would demonstrate a blind pouch of the proximal oesophagus and abdominal air. But our patient demonstrated prompt pulmonary aspiration through the tracheo-oesophageal fistula [6]. This fact might help us to discriminate the communication of the fistula with the proximal or distal oesophagus. Thus, the type of EA could be initially inferred from the X-ray study.

Most H-type fistulas can be approached from the right side of the neck because they are usually situated at or above the level of the second thoracic vertebra. In our case, the oesophagographic scan showed that the proximal end was at the third thoracic vertebra. The case could be operated thoracoscopically by experienced surgeons.

Although some H-type variations of EA have been described previously [7], to our knowledge, the presence of an H-type congenital tracheo-oesophageal fistula associated with oesophageal stenosis in a neonate has yet to be reported in the literature. Thus, surgeons treating patients with suspected EA must be aware of this variant. They should especially be prepared to perform prompt pulmonary aspiration during the oesophagram.

## Funding

No funding was secured for this study.

**Conflict of interest:** The authors have no conflicts of interest to disclose.

### Reviewer information

Interactive CardioVascular and Thoracic Surgery thanks Georges Decker, Rajashekara H.V. Reddy and the other, anonymous reviewer(s) for their contribution to the peer review process of this article.
